# Effect of Esaxerenone in Patients With Persistent Atrial Fibrillation and Hypertension After Catheter Ablation

**DOI:** 10.7759/cureus.91898

**Published:** 2025-09-09

**Authors:** Manabu Kashiwagi, Akio Kuroi, Natsuki Higashimoto, Satoshi Hata, Akira Taruya, Yasutsugu Shiono, Takashi Yamano, Takashi Tanimoto, Hironori Kitabata, Atsushi Tanaka

**Affiliations:** 1 Department of Cardiovascular Medicine, Wakayama Medical University, Wakayama, JPN

**Keywords:** brain natriuretic peptide, catheter ablation, esaxerenone, hypertension, persistent atrial fibrillation

## Abstract

Background

In this study, we investigated the effects of esaxerenone, a nonsteroidal mineralocorticoid receptor antagonist, in patients with persistent atrial fibrillation (AF) and hypertension after they had undergone catheter ablation.

Methodology

Our retrospective analysis included 157 consecutive patients with this condition. Overall, 40 patients received esaxerenone within three months after catheter ablation (hereafter, the esaxerenone group), and 117 of them did not (hereafter, the non-esaxerenone group). Other conventional pharmacologic agents were used similarly in the two groups.

Results

The rate of freedom from AF at one year was not significantly different between the two groups (80% (n = 32) vs. 84% (n = 98), p = 0.60). Independent predictors for AF recurrence according to univariate and multivariate Cox regression analysis were the duration of AF history (p < 0.01) and left ventricular myocardial index (LVMI) before ablation (p < 0.01). There was no significant difference in brain natriuretic peptide or LVMI before ablation (p = 0.43 and 0.89) between the groups. However, the esaxerenone group showed lower levels of brain natriuretic peptide at six months after catheter ablation (50.0 (16.3, 83.9) vs. 64.6 (25.9, 132.0) ng/mL, p = 0.02) and significant decrease in LVMI at one year after catheter ablation (-3.8 (-21.7, 3.5) vs. 0.0 (-4.3, 9.9) g/m^2^, p < 0.01) compared with the non-esaxerenone group.

Conclusions

Esaxerenone did not suppress the recurrence of AF in our cohort of patients with persistent AF and hypertension after catheter ablation. However, we suggest that esaxerenone is cardioprotective by reducing brain natriuretic peptide levels and LVMI.

## Introduction

Mineralocorticoid receptor antagonists (MRAs) are effective for resistant hypertension and heart failure with reduced ejection fraction [1]. However, conventional steroidal MRAs (spironolactone and eplerenone) have limited usefulness because of associated side effects, such as hyperkalemia and gynecomastia, and they are contraindicated for patients with moderate or severe renal dysfunction. Esaxerenone is a novel nonsteroidal MRA that may overcome the limitations of conventional MRAs [2]. To date, esaxerenone has only been used for hypertension. However, retrospective observational studies have suggested that esaxerenone might have an anti-heart failure effect by reducing brain natriuretic peptide (BNP) levels similar to conventional MRAs [3-5]. In addition to the anti-heart failure effects, an improvement in left ventricular myocardial mass volume was demonstrated in a prospective, randomized study in patients with hypertension and left ventricular hypertrophy [6]. Elsewhere, administration of esaxerenone reportedly reduced cardiac fibrosis and oxidative stress in rat-based studies [7].

Atrial fibrillation (AF) is related to the development of systemic embolism and heart failure. Catheter ablation for AF has made remarkable progress in recent years, and rhythm control therapy has been proven to suppress cardiovascular events [8]. However, the long-term recurrence rate after catheter ablation remains high, especially in cases of persistent AF [9]. Suppression of the renin-angiotensin-aldosterone system can reduce AF susceptibility, and conventional steroidal MRAs (spironolactone and eplerenone) have been shown to reduce AF recurrence after catheter ablation [10-12]. MRAs reduce the risk of new-onset AF or recurrence only in heart failure with reduced ejection fraction [13]. Finerenone, a new nonsteroidal MRA, reportedly has the potential to reduce the risk of new-onset AF in patients with chronic kidney disease and type 2 diabetes mellitus [14]. In the present study, we investigate whether esaxerenone, which has similarities to other MRAs, has a favorable effect after catheter ablation for persistent AF.

## Materials and methods

Study population

This retrospective, observational study included patients with persistent AF and hypertension who underwent catheter ablation at Wakayama Medical University Hospital between April 2018 and December 2023. We excluded cases without follow-up data one year after ablation; those with repeated catheter ablation for atrial arrythmia within one year after previous catheter ablation; those who had already been treated with MRA and angiotensin receptor neprilysin inhibitor before ablation; those who were newly administered with beta-blockers, sodium-glucose cotransporter-2 inhibitors, or renin-angiotensin-aldosterone system inhibitors within one year after ablation; and those with severe renal impairment (creatinine-based estimated glomerular filtration rate <30 mL/minute/1.73 m^2^). In patients who agreed to start esaxerenone administration, esaxerenone was orally administered once daily at an initial dose of either 1.25 or 2.5 mg/day within three months after catheter ablation. This study was conducted in accordance with the Declaration of Helsinki and was approved by the Wakayama Medical University Research Ethics Committee (approval number: 3557). The requirement for written informed consent was waived because of the retrospective nature of the study.

Ablation method

The specific strategies for catheter ablation for persistent AF were decided by individual operators. All patients were mildly sedated with dexmedetomidine hydrochloride and/or thiopental sodium.

For patients with radiofrequency ablation, a decapolar catheter was inserted into the coronary sinus, and a mapping catheter was placed in the pulmonary veins. A deflectable sheath was also introduced for an irrigated ablation catheter (SmartTouch SF, Biosense Webster, Irvine, CA, USA). Ipsilateral wide area circumferential pulmonary vein isolation under force time integral or ablation index guide was performed, but we occasionally considered additional radiofrequency ablation for non-pulmonary vein foci.

For patients with cryoballoon ablation, a decapolar catheter was inserted into the coronary sinus. A 15-F steerable sheath (FlexCath, Medtronic, Minneapolis, MN, USA) was introduced into the left atrium for insertion of a cryoballoon (Arctic Front Advance, Medtronic, Irvine, CA, USA) with a circular catheter (Achieve, Medtronic, Dublin, Ireland). Basically, 180-second freezing was performed at each pulmonary vein. In unsuccessful cases and those with non-pulmonary vein foci, additional radiofrequency ablation was occasionally performed.

Transthoracic echocardiography

A modified Simpson method was applied to calculate the left ventricular ejection fraction. E- and A-waves and deceleration time were measured by mitral inflow profile assessed in the apical four-chamber view using pulsed-wave Doppler echocardiography, with the sample volume placed at the tips of mitral leaflets during diastole. The e’ velocity from the septal and lateral mitral valve annulus was measured using Doppler tissue imaging of the mitral annulus in the apical four-chamber view. The mean E/e’ ratio was then calculated. Left ventricular mass was estimated using Devereux’s method [15]. Measurement for left ventricular mass was indexed by body surface area and defined as left ventricular mass index (LVMI).

Follow-up

Patients were scheduled for follow-up visits at three, six, and 12 months after the procedure, and a 12‐lead electrocardiogram was routinely performed at every visit. AF recurrence within the first three months after radiofrequency catheter ablation was considered to be transient, so a blanking period of three months was applied [12]. Laboratory blood tests were also performed at six months, and transthoracic echocardiography was performed at 12 months. If the patient had an episode of palpitations, further examinations, including Holter monitoring, were considered to determine atrial tachycardia.

Statistical analysis

Statistical analysis was performed using JMP Pro version 17.0 for Macintosh (SAS Institute, Cary, NC, USA). Results are expressed as median (interquartile range), and qualitative data as numbers and percentages. The nonparametric Mann-Whitney U test was used to test for differences between the two groups, Pearson’s chi-square test was used for categorical variables, and Kaplan-Meier log-rank test survival probability analysis was applied to the two different groups to evaluate AF recurrence. Cox proportional hazards regression analysis was performed to determine the independent predictors of AF recurrence. The risk of AF recurrence was expressed as a hazard ratio (HR), 95% confidence interval, and p-value. The receiver operating curve was used to determine the best cut-off value of duration of AF history and LVMI for AF recurrence. The best cut-off value was determined according to the maximum Youden’s index. P-values <0.05 were considered to be statistically significant.

## Results

Patient characteristics and ablation method

In total, 157 consecutive patients were retrospectively enrolled in this study, including 40 patients who received esaxerenone (hereafter, the esaxerenone group) and 117 patients who did not (hereafter, the non-esaxerenone group). Baseline clinical characteristics at admission are summarized in Table 1. There were no differences between the esaxerenone and non-esaxerenone groups in baseline characteristics or echocardiographic findings. Detailed catheter ablation procedures and medication records are presented in Table 2. Approximately 80% of patients underwent their first catheter ablation, and there was no difference between the two groups in AF or catheter ablation data. As an elevated K^+^ level was observed in one patient after esaxerenone treatment, their dose of esaxerenone was reduced from 2.5 mg to 1.25 mg.

**Table 1:** Baseline clinical characteristics. Data presented are median (quartiles) or N (%). ^*^: Mann-Whitney U test; ^**^: Pearson’s chi-square test. BMI: body mass index; LV: left ventricular; IVS: interventricular septum; PW: posterior wall

	Esaxerenone group	Non-esaxerenone group	Value	P-value
Patient, n	40	117		
Age, years	68 (64–75)	72 (68–75)	Z = -1.83	0.07^*^
Men	27 (68)	73 (62)	Χ^2^ = 0.34	0.56^**^
BMI, kg/m^2^	24.6 (22.0–26.6)	24.3 (22.7–27.1)	Z = -0.03	0.98^*^
Diabetes mellitus	8 (20)	16 (14)	Χ^2^ = 0.92	0.34^**^
Heart failure	11 (28)	21 (18)	Χ^2^ = 1.68	0.20^**^
Stroke	4 (10)	10 (9)	Χ^2^ = 0.08	0.78^**^
Duration of persistent atrial fibrillation, months	8 (3–23)	7 (5–12)	Z = 0.39	0.70^*^
Systolic blood pressure, mmHg	135 (112–146)	131 (120–144)	Z = -0.26	0.79^*^
Diastolic blood pressure, mmHg	80 (70–94)	80 (72–89)	Z = 0.16	0.88^*^
Serum potassium, mEq/L	4.3 (4.0–4.6)	4.3 (4.0–4.5)	Z = 0.02	0.98^*^
Estimated glomerular filtration rate, mL/minute/1.73m^2^	45.8 (40.0–56.1)	43.9 (36.6–52.6)	Z = 0.35	0.18^*^
LV diastolic dimension, mm	47 (43–54)	46 (43–51)	Z = 1.22	0.22^*^
LV systolic dimension, mm	33 (28–40)	31 (27–36)	Z = 1.42	0.15^*^
IVS, mm	9 (8–10)	9 (9–10)	Z = -0.07	0.94^*^
PW, mm	9 (8–10)	9 (8–10)	Z = -0.38	0.71^*^
Ejection fraction, %	55 (48–58)	56 (51–60)	Z = -1.34	0.18^*^
Left atrial diameter, mm	44 (40–46)	43 (39–46)	Z = 0.63	0.53^*^
Mean E/e’ ratio	9.1 (7.5–12.4)	10.2 (7.4–13.0)	Z = -0.75	0.46^*^

**Table 2:** Catheter ablation and medication data. Data presented are N (%). ^*^: Pearson’s chi-square test. ACE-I: angiotensin-converting enzyme inhibitor; ARB: angiotensin receptor blocker; SGLT2: sodium-glucose cotransporter-2

	Esaxerenone group (n = 40)	Non-esaxerenone group (n = 117)	Value	P-value
Catheter ablation				
Cryoballoon ablation	6 (15)	11 (9)	Χ^2^ = 0.97	0.33^*^
Pulmonary vein isolation	36 (90)	107 (91)	Χ^2^ = 0.08	0.78^*^
Left atrial posterior isolation	7 (18)	16 (14)	Χ^2^ = 0.35	0.55^*^
Cavotricuspid isthmus line	20 (50)	44 (38)	Χ^2^ = 1.90	0.17^*^
Other additional ablation method	3 (8)	5 (4)	Χ^2^ = 0.64	0.42^*^
Multiple sessions of catheter ablation	10 (25)	17 (15)	Χ^2^ = 2.30	0.13^*^
Medication at admission				
Calcium channel blocker	20 (50)	73 (62)	X^2^ = 1.90	0.17^*^
ACE-I/ARB	20 (50)	73 (62)	X^2^ = 1.90	0.17^*^
β-blocker	32 (80)	81 (69)	X^2 ^= 1.71	0.19^*^
SGLT 2 inhibitors	4 (10)	8 (7)	X^2^ = 0.42	0.52^*^
Anti-arrhythmic agent	5 (13)	7 (6)	X^2^ = 1.79	0.20^*^

Prognosis of AF recurrence

**Figure 1:**
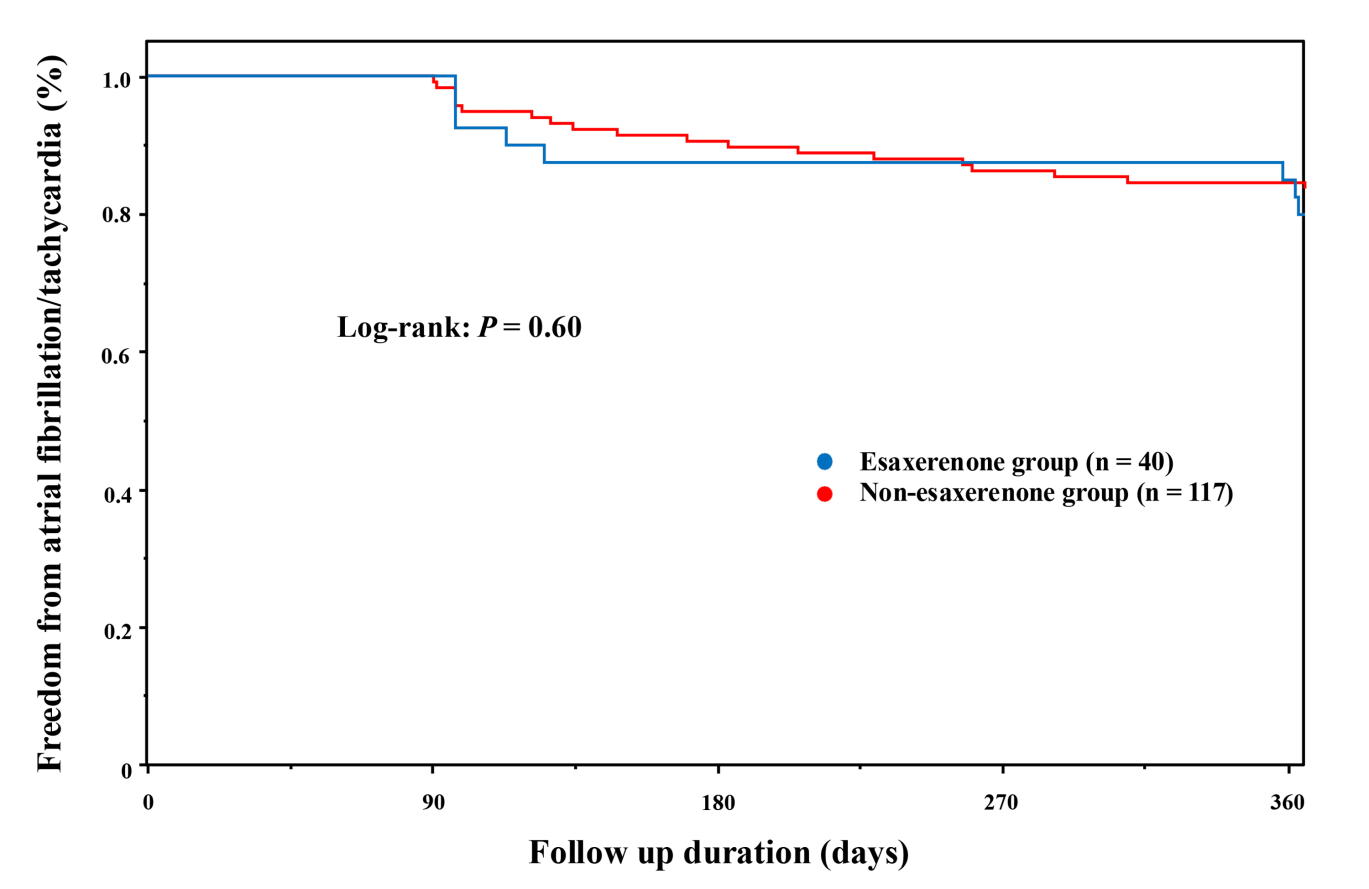
Kaplan-Meier curve of freedom from recurrence.

**Figure 2:**
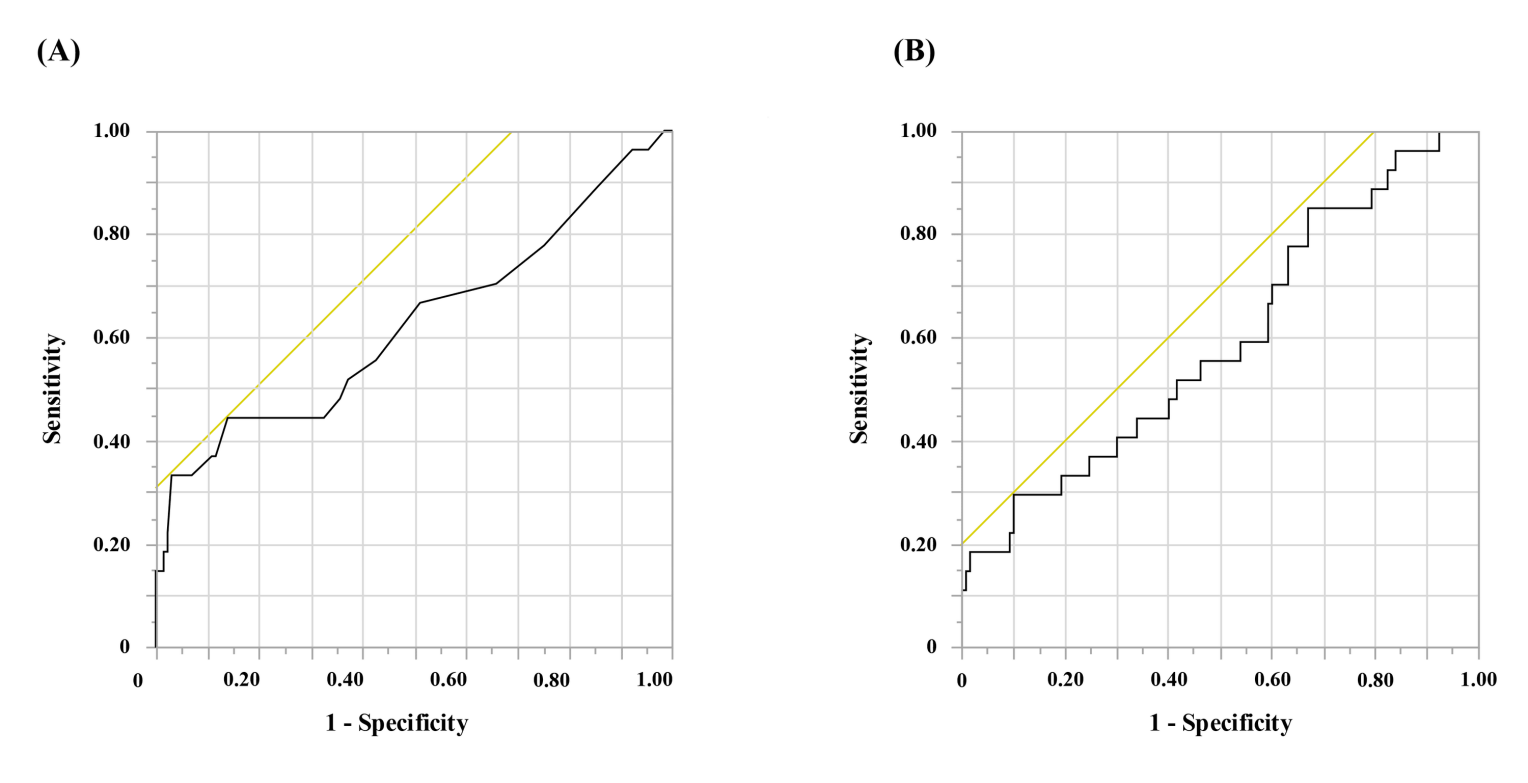
Receiver operating curve to predict recurrence. (A) Duration of atrial fibrillation recurrence. (B) Left ventricular mass index.

The freedom from AF rate at one year was 80% (n = 32) in the esaxerenone group and 84% (n = 98) in the non-esaxerenone group. Kaplan-Meier curves for the freedom from AF recurrence between the two groups are shown in Figure 1. The log-rank test showed no significant difference (p = 0.60). In this study, we excluded patients who underwent repeat ablation within one year after the previous AF ablation. No cardiovascular events occurred in any of the patients, including death, heart failure, myocardial infarction, or cerebral infarction. Univariate and multivariate Cox regression analysis revealed that the only independent predictors for AF recurrence were the duration of AF history (HR = 1.04 (1.02, 1.05), p < 0.01) and LVMI before catheter ablation (HR = 1.02 (1.01, 1.04), p < 0.01) (Table 3). From the receiver operating curve, the best cut-off vales of 18 months of AF duration (area under curve = 0.63, sensitivity = 44%, specificity = 86%, p < 0.01) and 136.0 g/m^2^ of LVMI (area under curve = 0.59, sensitivity = 30%, specificity = 90%, p = 0.02) were predictors of AF recurrence (Figure 2).

**Table 3:** Cox regression analysis for atrial fibrillation recurrence. BMI: body mass index; AF: atrial fibrillation; ACE-I: angiotensin-converting enzyme inhibitor; ARB: angiotensin receptor blocker; SGLT2: sodium-glucose cotransporter-2

Parameters	Univariate		Multivariate	
	Hazard ratio (95% CI)	P-value	Hazard ratio (95% CI)	P-value
Age	0.98 (0.94–1.04)	0.50	-	-
Men	1.65 (0.70–3.89)	0.24	-	-
BMI	1.00 (0.89–1.10)	0.95	-	-
Diabetes mellitus	0.66 (0.20–2.18)	0.47	-	-
Heart failure	0.66 (0.23–1.90)	0.41	-	-
Stroke	1.85 (0.64–5.34)	0.29	-	-
Duration of persistent AF	1.03 (1.02–1.05)	<0.01	1.04 (1.02–1.05)	<0.01
Systolic blood pressure	1.01 (0.99–1.03)	0.26	-	-
Diastolic blood pressure	1.00 (0.97–1.03)	0.88	-	-
Estimated glomerular filtration rate	1.01 (0.98–1.03)	0.88	-	-
Calcium channel blocker	1.01 (0.47–2.19)	0.97	-	-
ACE-I/ARB	1.73 (0.76–3.95)	0.18	-	-
β-blocker	0.76 (0.34–1.68)	0.50	-	-
SGLT2 inhibitors	0.43 (0.06–3.13)	0.33	-	-
Anti-arrhythmic agent	0.95 (0.22–4.00)	0.94	-	-
Esaxerenone group	1.25 (0.55–2.85)	0.61	-	-
Brain natriuretic peptide	1.00 (1.00–1.00)	0.39	-	-
Ejection fraction, %	1.00 (0.96–1.06)	0.87	-	-
Left atrial diameter, mm	1.04 (0.97–1.12)	0.28	-	-
Left ventricular mass index, g/m^2^	1.02 (1.00–1.03)	0.02	1.02 (1.01–1.04)	<0.01
Cryoballoon ablation	0.62 (0.14–2.61)	0.48	-	-
Multiple sessions of catheter ablation	1.14 (0.43–3.02)	0.79	-	-

BNP and echocardiographic parameters

**Figure 3:**
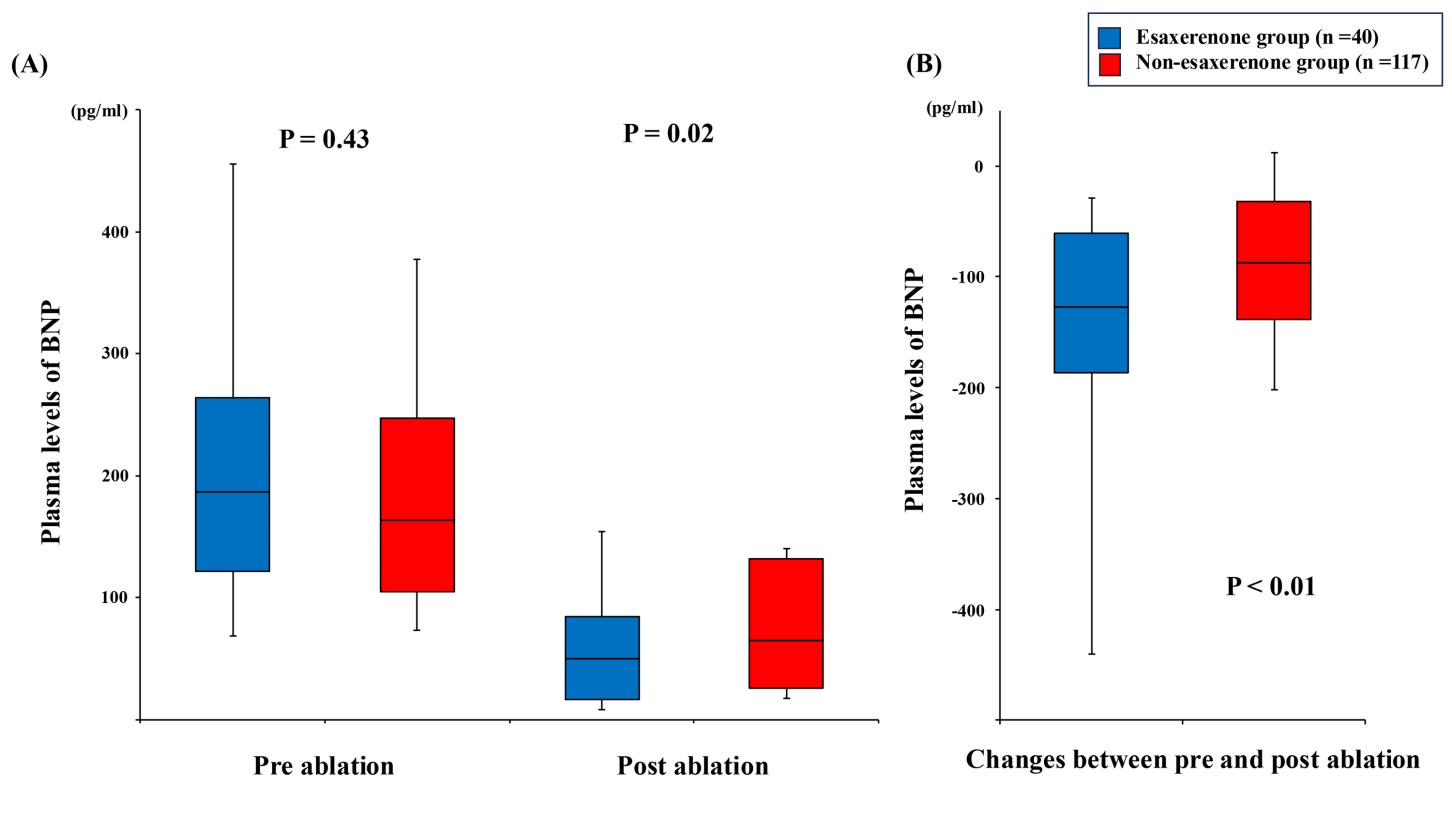
Serial changes of B-type natriuretic peptide levels. (A) Trends pre-catheter ablation and post-catheter ablation. (B) Changes between pre-catheter ablation and post-catheter ablation. Between-group comparisons were performed using the Mann-Whitney U test.

The serial change in BNP levels before and six months after catheter ablation is shown in Figure 3. There was no significant difference between the groups in BNP levels before ablation (186.2 (121.6, 264.0) vs. 163.3 (104.4, 246.6) ng/mL, p = 0.43). The esaxerenone group showed lower levels of BNP levels six months after catheter ablation (50.0 (16.3, 83.9) vs. 64.6 (25.9, 132.0) ng/mL, p = 0.02), and the change in BNP levels significantly decreased in the esaxerenone group compared with in the non-esaxerenone group (-127.1 (-186.3, -61.3) vs. -87.8 (-138.8, -31.8) ng/mL, p < 0.01). No patients had worsening of chronic kidney disease during the course of the study.

**Figure 4:**
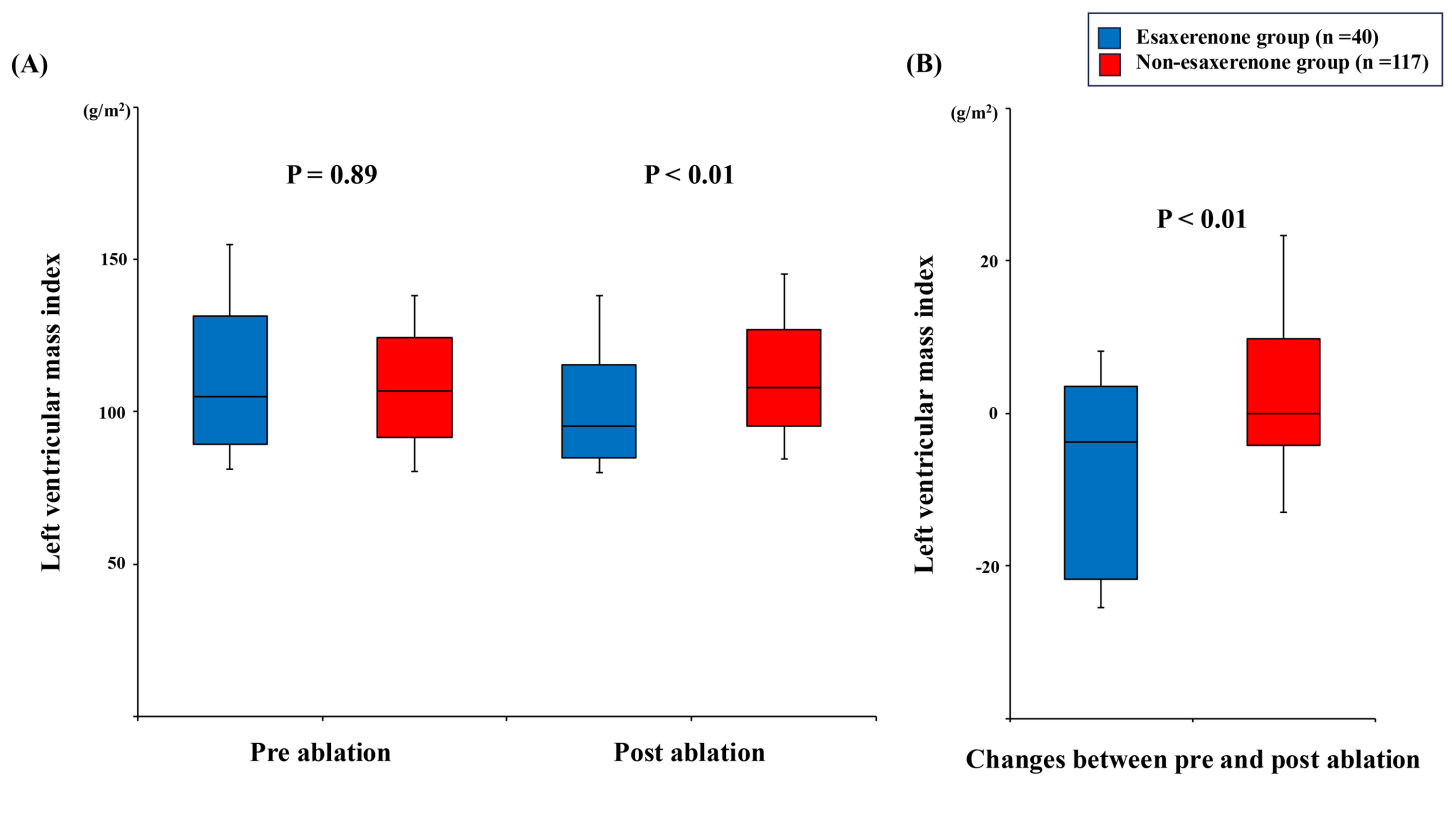
Serial changes of left ventricular mass index. (A) Trends pre-catheter ablation and post-catheter ablation. (B) Changes between pre-catheter ablation and post-catheter ablation. Between-group comparisons were performed using the Mann-Whitney U test.

Serial change in LVMI is shown in Figure 4. There was no significant difference in LVMI before ablation between the groups (104.9 (89.5, 131.3) vs. 106.9 (91.6, 124.3) g/m^2^, p = 0.89). The esaxerenone group showed lower LVMI one year after ablation (95.2 (85.0, 124.3) vs. 108.0 (95.2, 127.0), p < 0.01) and a significant decrease in LVMI (-3.8 (-21.7, 3.5) vs. 0.0 (-4.3, 9.9) g/m^2^, p < 0.01) compared with the non-esaxerenone group.

## Discussion

We investigated the effects of esaxerenone in patients with persistent AF and hypertension who underwent catheter ablation. There was no association between the recurrence rate of AF and the use of esaxerenone. However, patients treated with esaxerenone were demonstrated to have a significant decrease in BNP and LVMI according to echocardiography.

AF is commonly related to cardiovascular events, such as cerebral infarction and heart failure. Catheter ablation therapy for AF has been an established treatment that significantly suppresses the recurrence of AF compared with conventional drug therapy [16]. Cardiovascular events can also be suppressed by rhythm control therapy, including catheter ablation, compared with rate control therapy [8]. The recurrence rate of persistent AF remains high, despite the availability of pulmonary vein isolation by cryoballoon, hot balloon, or laser balloon (instead of radiofrequency ablation) and additional ablation beyond pulmonary vein isolation therapy. The addition of spironolactone to catheter ablation reportedly reduces the N-terminal of B-type natriuretic peptide and the size of the left atrium, and it suppresses the recurrence of AF compared to ablation alone [11]. Eplerenone was also proven in a retrospective observational study to suppress the recurrence of AF [12]. Finerenone reduced the new onset of AF in the FIDELIO-DKD study [13], but in the current study, no additional effect of esaxerenone in maintaining sinus rhythm was noted. This might be explained by the population of this study being small compared with those in previous reports. In addition, the quality of catheter ablation for AF has been improved by new technologies and strategies, and it is speculated that the contribution of the intervention effect of MRAs to suppress AF recurrence might be relatively decreased.

In this study, a remarkable decrease in BNP levels by esaxerenone was observed, which is consistent with the findings of previous retrospective observational studies and a prospective study [3-6]. As with the conventional MRAs (spironolactone and eplerenone), this study suggests that esaxerenone may have the potential to be suppressive for heart failure. The efficacy of finerenone for heart failure has been recently reported [17]. An increase in BNP levels has been reported as a predictor of AF recurrence after catheter ablation, so it is possible that esaxerenone may be shown to be effective in the suppression of AF recurrence in studies with larger populations and longer follow-up periods [18].

Echocardiography one year after catheter ablation revealed that the decrease in LVMI was larger in the esaxerenone group than in the non-esaxerenone group. In the ESES-LVH study (a multicenter, open-label, prospective, exploratory, interventional study that included patients with hypertension and left ventricular hypertrophy), esaxerenone reduced 9.9 g/m^2^ of LVMI. Esaxerenone was shown to reduce cardiac inflammation and oxidative stress in high-salt-loaded rats, leading to improved cardiac remodeling and reduced fibrosis [7]. Left ventricular hypertrophy and left atrial enlargement have been reported as predictors of recurrence after AF ablation [19]. Interestingly, our study suggests that LVMI is an independent predictor for AF recurrence after catheter ablation. Therefore, intervention in left ventricular hypertrophy with esaxerenone may contribute to preventing the recurrence of atrial fibrillation. However, further clinical and basic research is needed to elucidate the cardioprotective mechanism of esaxerenone.

Study limitations

This study has several limitations. As patients were retrospectively enrolled and were derived from a single center, there may be selection bias. The patient population was also relatively small. The terms of follow-up were short, and the patient populations in the two groups were different. Therefore, longer-term follow-ups and larger population studies are required for further elaboration. Moreover, we did not routinely use a Holter electrocardiogram for detecting AF recurrence, so recurrence of AF might be underdiagnosed. Furthermore, serial blood pressure data were not available. Finally, in patients on angiotensin receptor neprilysin inhibitors, evaluation of BNP may not be appropriate.

## Conclusions

Esaxerenone was not shown to suppress AF recurrence in our cohort of patients with persistent AF and hypertension after catheter ablation. However, esaxerenone seemed to be cardioprotective in terms of BNP level and LVMI reduction.
